# Modified transprepancreatic septotomy reduces postoperative complications after intractable biliary access

**DOI:** 10.1097/MD.0000000000009522

**Published:** 2018-01-05

**Authors:** Henggao Zhong, Xiaohong Wang, Lihua Yang, Lin Miao, Guozhong Ji, Zhining Fan

**Affiliations:** a The Medical Center for Digestive Diseases, The Second Affiliated Hospital of Nanjing Medical University; b Digestive Endoscopy Center, the First Affiliated Hospital with Nanjing Medical University & Jiangsu Province Hospital, Nanjing, China.

**Keywords:** cholangiopancreatography, complication, endoscopic retrograde, pancreatic stent, transprepancreatic septotomy

## Abstract

This study aimed to assess the clinical value of transprepancreatic septotomy indwelling guide wire or pancreatic duct stent in intractable endoscopic retrograde cholangiopancreatography (ERCP) for bile duct cannulation.

Of the 2107 patients treated by ERCP, a total of 81 cases with difficult bile duct cannulation underwent transprebiliopancreatic septotomy (referred to as the septotomy group, 37 cases) and transprepancreatic septotomy with pancreatic duct stent (modified septotomy group, 44 cases). Success rates of cannulation and postoperative complications for both methods were compared.

Among them, 77 cases were successfully administered bile duct cannulation. The success rates of the septotomy and modified septotomy groups were 91.89% and 97.73%, respectively, with no significant difference (*P* = .489). Of the 77 patients, 12 cases had complications. The septotomy group included 7 acute pancreatitis, 1 bleeding, and 1 biliary tract infection cases; while in the modified septotomy group, there were 1 acute pancreatitis, 1 bleeding, and 1 biliary tract infection cases. The occurrence rate of acute pancreatitis in the modified septotomy group was lower than that of the septotomy group (2.33% vs 20.59%) with a significant difference (*P* = .026).

These findings indicate that transprepancreatic septotomy with pancreatic duct stent seems to be a safe and feasible operation with reducing complication rates.

## 1 Introduction

**Figure 1:**
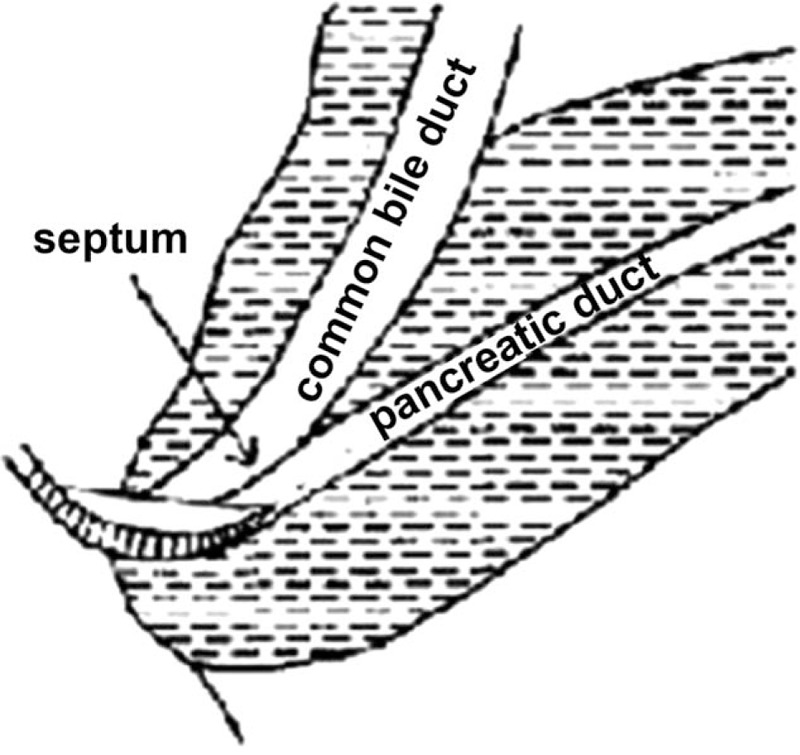
Septum between the common bile duct and pancreatic duct.

The success of common bile duct cannulation is key to endoscopic retrograde cholangiopancreatography (ERCP). However, due to the minor duodenal papilla, anatomical factors (such as papilla hardening, softness, and diverticulum), Billroth II gastrectomy, and anastomosis, selective cannulation is not easily successful. Despite the use of various tubes and guide wires for cannulation, the failure rate of bile duct cannulation remains high.^[1]^ Endoscopic needle-knife precut papillotomy significantly improves the success rate of ERCP-based bile duct cannulation, but the rate of complications reaches 6% to 20%.^[2]^ There is a thin septum between the pancreatic duct and common bile duct (Fig. 1), and bile duct cannulation difficulty may be related to bile duct being blocked by the septum. We used an arch knife to cut the septum through the pancreatic duct towards the bile duct.^[3]^ In our previous study, we found that the success rate of bile duct cannulation of transprepancreatic septotomy and needle-knife precut papillotomy was the same; however, transprepancreatic septotomy is safer than precut papillotomy.^[4]^ Placement of pancreatic stents is an increasingly adopted approach to reduce the risk of post-ERCP complications. Herein, we compared transpancreatic septotomy (referred to as the septotomy group) with transprepancreatic septotomy with pancreatic duct stent (modified septotomy group), and assessed common bile duct cannulation success and complication occurrence rates, to identify the best cannulation method.

## 2 Materials and methods

### 2.1 Clinical data

**Figure 2:**
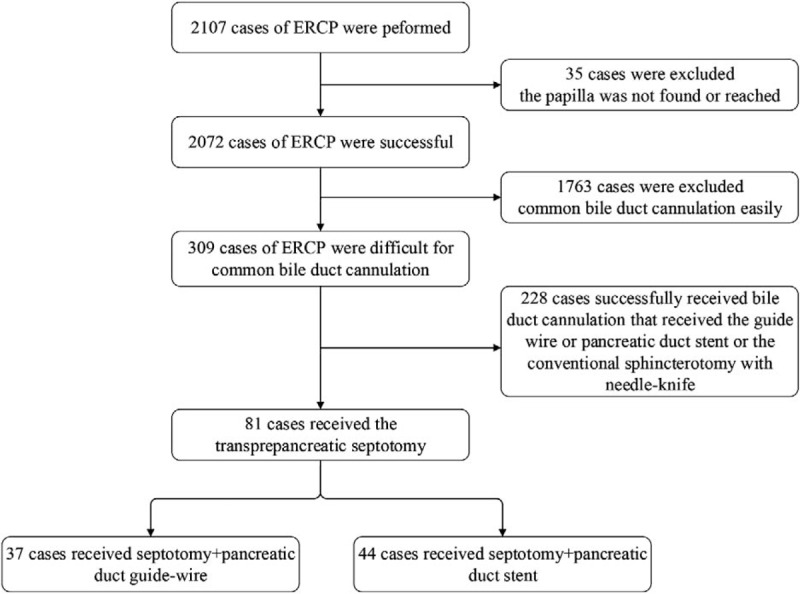
The flow chart of septotomy group/modified septotomy group including and excluding.

From June 2012 to June 2014, the digestive medical center at our hospital planned 2107 ERCP. Except for 35 cases in whom the papilla was not found or reached, including Billroth II postoperation, Roux-en-Y postoperation, and duodenal stenosis, 2072 cases of ERCP were successful. A total of 309 cases of ERCP with difficult common bile duct cannulation received a guide wire or plastic trestle in the pancreatic duct, or underwent conventional sphincterotomy with needle-knife; 228 cases successfully underwent bile duct cannulation, while 81 cases failed and received transprepancreatic septotomy (septotomy group, 37 cases) or transprepancreatic septotomy with pancreatic duct stent (modified septotomy group, 44 cases). Cannulation success and complication occurrence were compared for both methods (Fig. 2). Our study was approved by the Institutional Review Board of Nanjing Medical University. After obtaining written informed consent to participate from each patient or from patients’ representatives, we collected research data from medical records.

### 2.2 Equipment

Side-view duodenoscope (Olympus TJF-260V Duodenoscope), sphincterotomy needle-knife and arch knife (Boston Scientific Corporation or Cook Medical Incorporation), and high-frequency generator (ERBE Elektromedizin GmbH ICC 80) were used for the study.

### 2.3 Operation method

**Figure 3:**
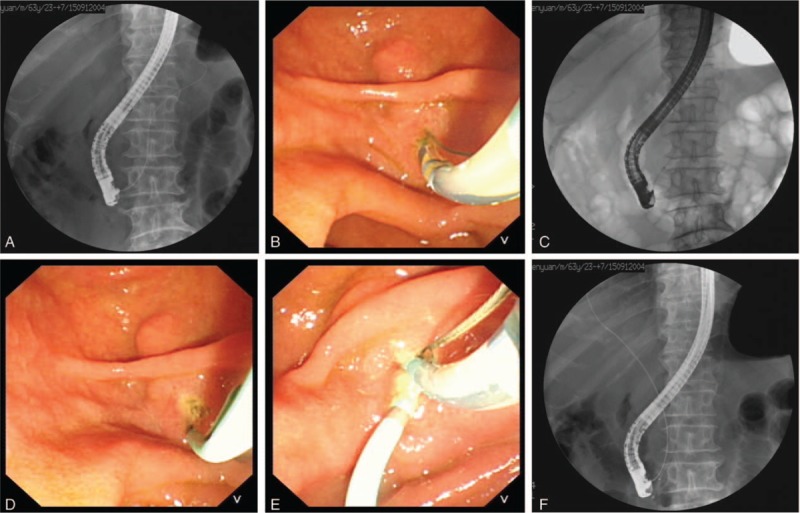
Transprepancreatic pre-septotomy via the pancreatic duct. (A) The guide wire entered the pancreatic duct; (B) The septum was cut from the pancreatic duct toward the bile duct; (C) Pancreatic duct stenting; (D) After pancreatic duct stenting, (E) Cannulation toward the pancreatic duct was repeated, (F) Bile duct cannulation was successful.

An endoscope was inserted until it reached the duodenal papilla. The patients in whom the guide wire could enter the pancreatic duct but not the bile duct (more than three time) were treated as follows: Transprepancreatic septotomy: if the guidewire repeatedly entered the pancreatic duct, and it was left in the pancreatic duct. A bow knife was then inserted into the pancreatic duct to make a small incision (<5 mm) into the papilla toward the bile duct. The transprepancreatic septum was cut, and bile duct cannulation was performed. Transprepancreatic septotomy with pancreatic duct stent: After the arch knife was inserted into the pancreatic duct, the papilla was cut toward the bile duct (<5 mm). The transprepancreatic septum was cut and an internal stent was placed within the pancreatic duct, and then, bile duct cannulation was performed (Fig. 3).

### 2.4 Diagnostic criteria of postoperative ERCP complications

The severity of acute pancreatitis is classified mild, moderate, or severe based on consensus criteria. Mild pancreatitis is defined as serum amylase at least 3 times normal at more than 24 hours after the procedure, and is not associated with complications or organ dysfunction and recovery is uneventful. In contrast, severe pancreatitis is characterized by pancreatic dysfunction, local and systemic complications, and required intervention (percutaneous drainage or surgery).^[5]^ Infection: within 24 hours after ERCP, the right upper abdomen was painful, accompanied by fever >38.5°C, as well as white blood cell count >10.0 × 109/L with no other infectious lesions. Alimentary tract hemorrhage was defined as clinical evidence of bleeding, including hematemesis, melena, or hemoglobin reduced by >5% of normal level within 24 hours after ERCP.^[6]^ Perforation: subcutaneous emphysema, retroperitoneal gas shadow, or subphrenic free air after ERCP.

### 2.5 Statistical analysis

The Statistical Product and Service Solutions SPSS 15.0 statistical software and χ^2^ test were used to assess success rates of cannulation and complications. *P* < .05 was considered statistically significant.

## 3 Results

### 3.1 Success rate of bile duct cannulation

The septotomy group included 37 cases, with 22 males and 15 females, aged 21 to 83 years (average age of 55.9 years). In the modified septotomy group, there were 44 cases, including 25 males and 19 females, aged 19 to 86 years (average age of 57.7 years). There were no significant differences in pathogeny distribution and duodenal papilla morphology between the 2 groups. A total of 77 cases out of 81 successfully underwent bile duct cannulation. The success rate of the septotomy group was 91.89% (34/37), while that of the modified septotomy group was 97.73% (43/44), with no significant difference (*P* > .05) (Table 1).

**Table 1:**
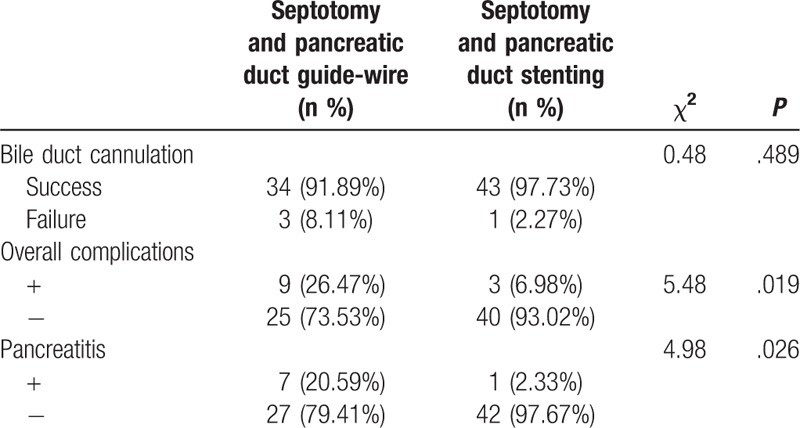
The bile duct cannulation success rates and postoperative complication rates between the 2 groups.

### 3.2 Postoperative complications

A total of 12 cases out of 77 had complications, including 8 acute pancreatitis (no severe pancreatitis), 2 bleeding, and 2 biliary tract infection cases. Among them, 7 acute pancreatitis, 1 bleeding, and 1 biliary tract infection cases were in the septotomy group, while the modified septotomy group included 1 acute pancreatitis, 1 bleeding, and 1 biliary tract infection cases. The total occurrence rate of complications in the modified septotomy group was significantly lower than that of the septotomy group (6.98% vs 26.47%; *P* = .019) (Table 1). The occurrence rate of acute pancreatitis in the modified septotomy group was significantly lower than that of the septotomy group (2.33% vs 20.59%; *P* = .026) (Table 1). Those patients with failed ERCP underwent percutaneous transhepatic choledochus drainage (PTCD) or surgery. Patients with acute pancreatitis recovered after 1 week with fasting, anti-infection, and inhibition of pancreatic secretion. Patients with perioperative hemorrhage received 1:10,000 aqueous epinephrine via the thick papillary mucosa, which stopped bleeding, as well as hemostatics after the operation by intravenous infusion. Patients with biliary tract infection were healed after 3 to 5 days of anti-infective therapy.

## 4 Discussion

The success of ERCP selective bile duct cannulation is key to subsequent biliary tract disease treatment. Several technical measures have been evaluated and proven to be successful in increasing the success rate of bile duct cannulation, such as guide wire, needle-knife precut papillotomy.^[7]^ In the actual operation, the success rate of pancreatic duct cannulation is common, and this may be related to bile duct blocking by the ampulla septum. The septum lies between the pancreatic duct and common bile duct. We previously applied transprepancreatic dissection through the pancreatic duct, and found that it further increases the success rate of cannulation.^[3]^ This effect is related to pancreatic duct blockage by the guide wire or stent, which facilitates the second guide wire entering the bile duct.^[8]^ We believe the direction change of bile duct axis with the guide wire or stent is also involved, as well as bile duct linearization. However, the occurrence rate of complications in this method was up to 8.3%, and postoperative pancreatitis, hemorrhage, and biliary tract infection were observed.

In this study, we retrospectively evaluated outcomes pertaining to ERCP complications of transprepancreatic septotomy leaving the guide wire (septotomy group) and pancreatic duct stent (modified septotomy group). Both the 2 groups had high success rates, while the latter technology is safer because of its lower plication rate. Of course, both techniques must be carried out by skilled ERCP surgeons. Transprepancreatic septotomy through the pancreatic duct was first reported by Goff,^[9]^ with a total success rate of 97.5% in his long-term follow-up of 51 patients. A randomized study by Catalano et al^[10]^ demonstrated that transprepancreatic septotomy through the pancreatic duct had a higher success rate of bile duct cannulation; moreover, the complication rate of this method (3.4%) was lower than that of the needle-knife cannulation (11.8%). Therefore, endoscopists continually improved technology to improve the success rate of bile duct cannulation and prevent post-ERCP complications.

In our study, the pancreatic stent was placed in cases that initial cannulation was difficult, and these patients underwent bile duct cannulation, which could significantly reduce the incidence of postoperative complications, especially pancreatitis. As shown above, there was only 1 pancreatitis case in the modified septotomy group, which was significantly less than in the conventional septotomy group, and might be related to the protective effect of the pancreatic duct stent. Many studies have reported that pancreatic duct stenting could prevent postoperative pancreatitis, likely because the pancreatic duct stent ensures a smooth drainage of pancreatic juice.^[11,12]^

Occurrence of pancreatitis in the septotomy group was significantly higher than that of the modified septotomy group, indicating that the pancreatic duct stent plays an important role in preventing postoperative pancreatitis. Insertion of the guide wire into the pancreatic duct is a risk factor for postoperative pancreatitis. Nakai et al^[13]^ reported that entry of the guide wire into the pancreatic duct increases the incidence of postoperative pancreatitis from 5.3% to 13.0%, compared to without guidewire insertion. This might be related to pancreatic duct acinus damage caused by the guide wire.

Some limitations were pointed out as follows. This was a retrospective, observational study; a prospective randomized study with a larger sample should be conducted to investigate the efficacy of transprepancreatic septotomy with pancreatic duct stent. Pilot study data suggested a possible role for aggressive intravenous fluid resuscitation in preventing post-ERCP pancreatitis.^[14]^ Due to the small sample size of pancreatitis, we did not assess whether aggressive peri-procedural hydration reduced the incidence of pancreatitis following ERCP.

In conclusion, both modified and conventional transprepancreatic septotomy procedures have high success rate of ERCP bile duct cannulation, and the former method displays less complications and higher safety.
